# Benefits of melatonin on mortality in severe-to-critical COVID-19 patients: A systematic review and meta-analysis of randomized controlled trials

**DOI:** 10.1016/j.clinsp.2025.100638

**Published:** 2025-04-05

**Authors:** Jinlv Qin, Guizuo Wang, Dong Han

**Affiliations:** a Radioimmunoassay Center, Shaanxi Provincial People's Hospital, Xi'an, China; b Department of Respiratory and Critical Care Medicine, Shaanxi Provincial People's Hospital, Xi'an, China

**Keywords:** Mortality, Melatonin, COVID-19, Meta-analysis

## Abstract

**Objective:**

This meta-analysis aimed to determine the efficacy of melatonin on mortality in patients with severe-to-critical illness COVID-19.

**Methods:**

A systematic search was made of PubMed, Embase, Cochrane Library, and clinicaltrials.gov, without language restrictions. Randomized Controlled Trials (RCTs) on the treatment of severe-to-critical COVID-19 with melatonin, compared with placebo or blank, were reviewed. Studies were pooled to Odds Ratios (ORs), with 95 % Confidence Intervals (95 % CIs).

**Results:**

Three RCTs (enrolling 451 participants) met the inclusion criteria. Melatonin showed a significant effect on in-hospital mortality (OR = 0.19, 95 % CI 0.05 to 0.74; *p* = 0.02).

**Conclusions:**

Melatonin significantly reduced in-hospital mortality in patients with severe-to-critical COVID-19. Melatonin should be considered for severe-to-critical COVID-19 patients.

## Highlights

• This study was designed to assess melatonin in severe-to-critical COVID-19.
• Melatonin significantly reduced in-hospital mortality.
• Melatonin should be considered for severe-to-critical COVID-19 patients.

## Introduction

The Coronavirus Disease-2019 (COVID-19) pandemic is the worst pandemic in more than 100 years, causing numerous infections and deaths worldwide.^1^ Despite the use of multiple drugs with different mechanisms, mortality from COVID-19 remains high, especially in patients with Acute Respiratory Distress Syndrome (ARDS), sepsis, and associated Cytokine Release Syndrome (CRS).^2^^,^^3^ Most patients with COVID-19 develop an overactivated nonspecific immune response mediated by proinflammatory cytokines and chemokines, which play an important role in disease progression. Melatonin is a multifunctional hormone secreted by the pineal gland with antioxidant, anti-inflammatory, and immunomodulatory properties. Melatonin is involved in the regulation of sleep and blood pressure and has antiviral activity.^4^ Several Randomized Controlled Trials (RCTs) have assessed the effects of melatonin in COVID-19 patients, and their results differed. The effect of melatonin on mortality in patients with severe-to-critical COVID-19 was not clear.

Therefore, the aim of this study was to perform a meta-analysis of RCTs to determine the efficacy of melatonin on mortality in patients with severe-to-critical COVID-19.

## Methods

### Data sources and search strategy

This meta-analysis was based on the Preferred Reporting Items for Systematic Reviews and Meta-Analyses (PRISMA) statement.^5^ The protocol was previously registered in September 2023 in the PROSPERO database (Review register: CRD42023466646). PubMed, Embase, Cochrane Library, clinicaltrials.gov, and Google Scholar (for gray literature) were searched for studies up to September 2023, and updated in October 2024.

### Study selection

To be eligible for inclusion in the meta-analysis studies had to meet the following criteria: (a) Inclusion of hospitalized severe-to-critical COVID-19 patients; (b) Use of a randomized controlled design to make a comparison of melatonin with placebo or blank; and (c) Follow-up to observe the mortality. The search strings used for the databases were (“COVID-19″ OR “SARS-CoV-2″ OR “SARS-CoV-19″ OR “novel coronavirus 2019″ OR “novel coronavirus pneumonia”) AND “melatonin”. The reference lists of any relevant review articles were also screened to identify studies that might have been missed in this search. No language restrictions were applied to the study selection process.

### Data extraction and quality assessment

Two reviewers independently screened articles according to the inclusion criteria. The reviewers compared selected studies and differences were resolved by consensus. Data tables were used to collect all relevant data from texts, tables and figures of each included trial, including author, year of publication or last update posted, patient number and age, treatment category, and outcome, i.e., in-hospital mortality. Study quality was assessed using the Detsky Quality Assessment Scale.^6^^,^^7^ This is a 20-point scale for studies with statistically significant results and a 21-point scale for studies without statistically significant results.

### Risk of bias of included trials

Two reviewers independently assessed the risk of bias using the Cochrane Collaboration risk of bias tool for RCTs.^8^

### Data synthesis and statistical analysis

Meta-analyses were conducted where applicable; otherwise, outcomes were presented in narrative form. Data were analyzed using the RevMan Version 5.1 (The Cochrane Collaboration). Next, Odds Ratios (ORs) for discontinuous outcomes with corresponding 95 % Confidence Intervals (CIs) were computed for individual trials. Chi-Squared and Higgins I2 tests were used to assess heterogeneity among included trials. The authors used both a random-effects model and a fixed-effects model. A p-value < 0.05 was taken to indicate statistical significance. The p-value of Egger's linear regression test^9^^,^^10^ and Begg's rank correlation test^11^^,^^12^ (STATA version 12.0) were used to assess the presence of publication bias.

To assess the robustness of the results, meta‐regression analyses (STATA version 12.0) were carried out for sensitivity analysis to test the influence of potential effect modifiers such as sample size, sex, placebo or not, dosage of melatonin, and country.

## Results

### Study selection and characteristics

**Fig. 1:**
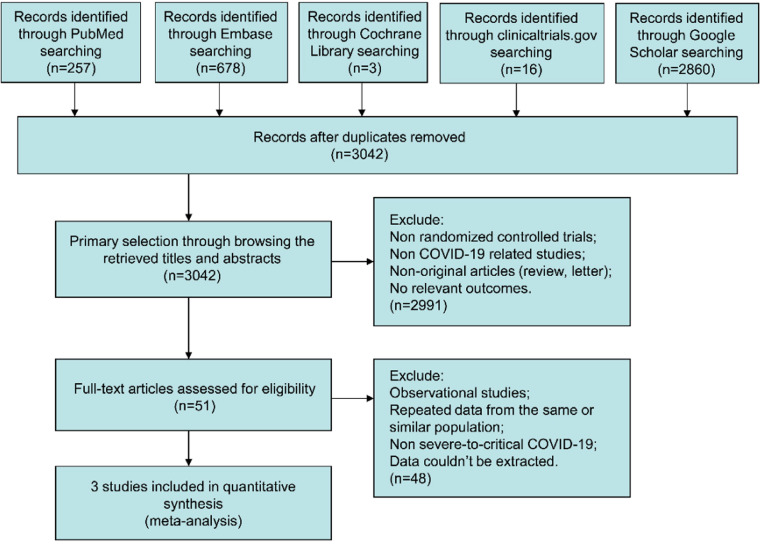
Flow chart for selection of studies.

**Fig. 2:**
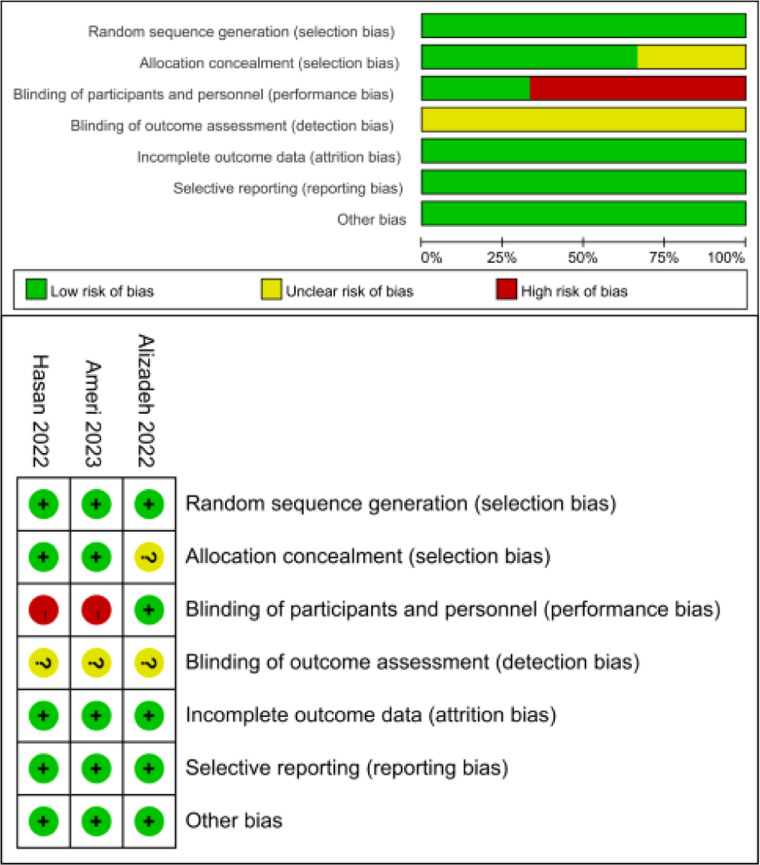
Assessment of the risk of bias for included RCTs.

Of 3814 trials recognized by the initial search, 51 were retrieved for more detailed assessment, and 3 trials^13^, ^14^, ^15^ were included in this meta-analysis (Fig. 1). Further details of the progress are described within the PRISMA flow diagram (Fig. S1). Baseline characteristics of trials included in this meta-analysis are shown in Table 1. A total of 451 patients were included: 224 assigned to the melatonin treatment groups and 227 to the control groups. The risk of biased results is summarized in Fig. 2.

**Table 1:** Baseline characteristics of trials included in meta-analysis.

Study (Ref. #)	Year	Quality Score	Regimen/day	n	Age, years (SD)	Male ( %)	Diabetes ( %)	Hypertension ( %)
Alizadeh^13^	2022	16	Melatonin 21 mg d1‒5	33	61.3 (18.1)	58	NR	NR
			Placebo	34	65.4 (19.3)	71	NR	NR
Ameri^14^	2023	16	Melatonin 10 mg d1‒7	109	54.6 (11.5)	43	33	31
			Standard of care	117	54.7 (13.4)	42	26	22
Hasan^15^	2022	18	Melatonin 10 mg d1‒14	82	56.8 (7.5)	71	31	61
			Standard of care	76	55.7 (8.0)	74	29	45

NR, Not Reported; SD, Standard Deviation.

### In-hospital mortality

**Fig. 3:**
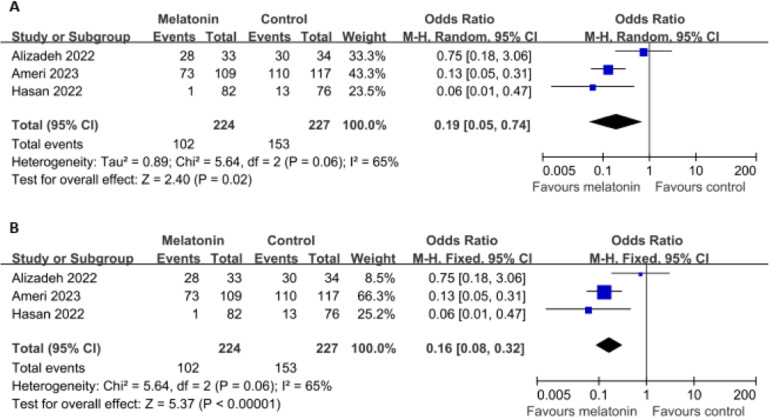
Forest plots assessing the efficacy of melatonin on (A) in-hospital mortality using a random-effects model, and (B) in-hospital mortality using a fixed-effects model.

Data on in-hospital mortality were available from three RCTs (including 451 patients). Melatonin showed a significant effect on in-hospital mortality, both in a random-effects model (OR = 0.19, 95 % CI 0.05 to 0.74; *p* = 0.02 [Fig. 3A]), and in a fixed-effects model (OR = 0.16, 95 % CI 0.08 to 0.32; *p* < 0.00001 [Fig. 3B]), with a rate of 45.54 % vs. 67.40 %. There was significant heterogeneity (I^2^ = 65 %; *p* = 0.06). Egger's test (*p* = 0.665) and Begg's test (*p* = 0.602) did not show evidence of publication bias.

### Sensitivity analysis

The present results were mostly confirmed when potential effect modifiers were introduced as covariates in the meta‐regression analysis. In this analysis, no significant impact was found on in-hospital mortality (Table 2).

**Table 2:** Potential effect modifier with change in tau^2^ and statistical significance for each outcome.

In-hospital mortality	Change in Tau^2^	p-value
Sample size	−0.19	0.878
Men	−0.67	0.623
Placebo	−0.66	0.627
Country	−2.32	0.259
Dosage	0.66	0.627

## Discussion

This meta-analysis is designed specifically to evaluate the efficacy of melatonin on in-hospital mortality in hospitalized patients with severe-to-critical COVID-19. Based on the present results, the authors observed that the use of melatonin was associated with a significant reduction in-hospital mortality.

COVID-19 is caused by SARS-CoV-2 and has caused a global pandemic. Although most patients have mild symptoms, mortality remains high in severe-to-critical illness patients, which may be largely attributable to an overactive immune response rather than the viral infection itself.^16^ This excessive inflammatory response, commonly referred to as Cytokine Storm Syndrome (CSS) or CRS, manifests as high fever, elevated CRP and ferritin levels, and cytopenias^17^^,^^18^ that can lead to lung damage, ARDS, Multiple Organ Dysfunction Syndrome (MODS), sepsis, and ultimately death.^19^, ^20^, ^21^

The use of melatonin as an adjunctive therapy has significantly reduced C-Reactive Protein (CRP) and improved clinical status in neonates with sepsis.^22^ Melatonin is a potent free radical scavenger that up-regulates antioxidant enzymes (such as glutathione peroxidase or superoxide dismutase) and down-regulates pro-oxidant enzymes.^13^ Melatonin can act on the enzyme thymidine kinase by membrane receptors or other receptors, such as two retinoid Z receptor subtypes (RZR α and β) and the three variants of other retinoid-related receptors (ROR α1, α2, and α3), which are related to the inflammatory response.^23^ Melatonin also confers anti-inflammatory effects on the cardiovascular system.^24^ Mechanical ventilation in critically ill patients is necessary but may pose a risk due to oxidative stress, which can be mitigated by melatonin.^25^ Melatonin increases the production of anti-inflammatory cytokines such as Interleukin (IL)-10^26^ and enhances humoral and cell-mediated responses.^4^ By enhancing humoral immunity, melatonin inhibits multiple inflammatory pathways (e.g., IL-1, IL-6, IL-8, and tumor necrosis factor α) and exerts antiviral effects.^4^^,^^27^ These inflammatory effects are directly related to the pulmonary and cardiovascular damage caused by severe COVID-19.^28^

Nevertheless, the effect of melatonin on mortality in patients with COVID-19 remains controversial. Two previous meta-analyses,^29^^,^^30^ both of which included only RCTs, found that melatonin was not associated with reduced mortality in patients with COVID-19. The present results differ from most other meta-analyses, which may be partly due to the inclusion of only severe-to-critically ill patients and partly due to the inclusion of very recent trials. Future large RCTs are needed to clarify the effective dose of melatonin, identify specific groups of beneficiaries, and explore the effectiveness of combination therapies.

This study met most of the methodological criteria recommended for systematic reviews and meta-analyses.^31^ However, some limitations need to be considered when interpreting the results of this study. Firstly, one included trial had a small sample size, which may have reduced the power of the results. Secondly, the number of included studies was small. Thirdly, two included trials did not include a placebo group. Fourthly, the dose of melatonin varied between studies. Finally, this meta-analysis was not patient-level, so the results should be considered provisional.

## Conclusions

Melatonin significantly reduced in-hospital mortality in patients with severe-to-critical COVID-19. Melatonin should be considered for severe-to-critical COVID-19 patients.

Data Availability Statement

Extracted data are available on request to the corresponding author.

## Ethics approval and consent to participate

Not applicable.

## Consent for publication

Not applicable.

## Authors' contributions

All authors, led by D.H., were involved in the concept and protocol design of the meta-analysis. J.Q. and G.W. screened the titles and abstracts and extracted data from the articles. J.Q. was primarily responsible for statistical analyses. G.W. was primarily involved in the interpretation of the quality data. All authors contributed to interpreting the results. J.Q. and D.H accessed and verified the data. All authors contributed to the writing of the article and approved its submission. D.H. was responsible for the decision to submit the article.

## Funding

None.

## Conflicts of interest

The authors declare no conflicts of interest.

## Appendix Supplementary materials

Image, application 1
