# Supplementary figures and images for: Benefits of melatonin on mortality in severe-to-critical COVID-19 patients: A systematic review and meta-analysis of randomized controlled trials
Source: Clinics (Sao Paulo). 2025 Apr 5;80:100638. doi: 10.1016/j.clinsp.2025.100638 (PMC12002743; doi:10.1016/j.clinsp.2025.100638)

**CLINICS-D-24-01061_ Supplementary Material**

**Supplementary Figure S1** PRISMA-2020 flow diagram.


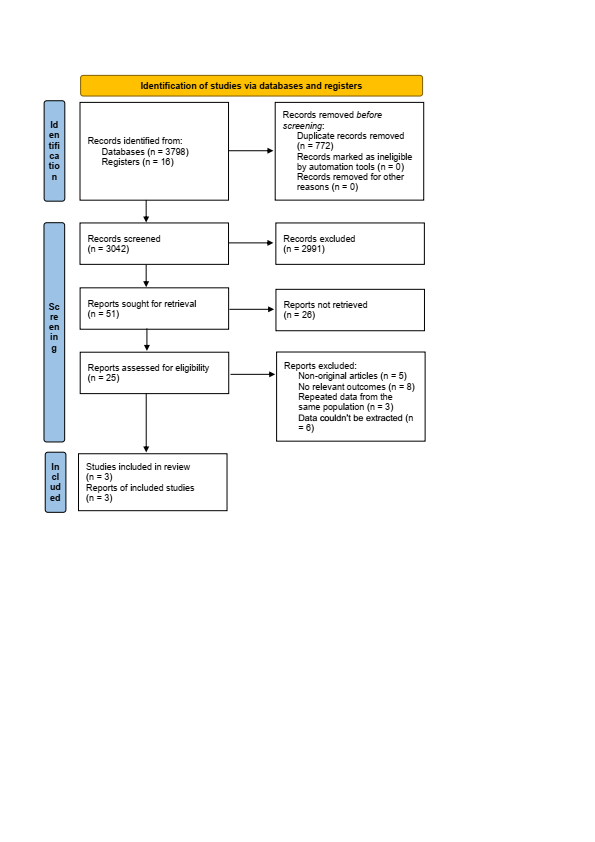

Supplement: Supplementary file 1 [file mmc1.docx]
